# Assessment of Dietary Under-Reporting in Italian College Team Sport Athletes

**DOI:** 10.3390/nu11061391

**Published:** 2019-06-21

**Authors:** Cinzia Ferraris, Monica Guglielmetti, Claudia Trentani, Anna Tagliabue

**Affiliations:** Human Nutrition and Eating Disorders Research Centre, Department of Public Health, Experimental and Forensic Medicine; University of Pavia, 27100 Pavia, Italy; monica.guglielmetti01@universitadipavia.it (M.G.); claudia.trentani@unipv.it (C.T.); anna.tagliabue@unipv.it (A.T.)

**Keywords:** dietary Intake, misreporting, under-reporting, athletes, team sport

## Abstract

Background: Nutrition is an important factor for sports performance and the assessment of dietary intakes in athletes can correct unhealthy eating habits. However, dietary assessment may be biased due to misreporting. The aim of our study was to investigate the occurrence of misreporting in a sample of collegiate team sport athletes. Methods: A total of 50 athletes participated. Each athlete filled in food records for seven days. Reported energy intake (EI) was considered in relation to the predicted basal metabolic rate (BMR) and expressed as the ratio EI/ BMR. All participants with EI/BMRestd ≤1.23 were classified as “low energy reporters” (LER), and those with an EI/BMRestd ratio >1.23 were classified as “adequate energy reporters” (AER). Results: According to cut-off values for under-reporting, 28 out of 50 athletes (56%) were classified as LER. The LER (16 M/12 F) had significantly higher BMI (23.17 ± 3.46 kg/m^2^ compared to 21.41 ± 1.91 kg/m^2^; *p* = 0.038) than the AER. The EI/kg fat free mass (FFM) was significantly lower in LER than the AER (33.34 ± 6.56 kcal/FFM compared to 48.51 ± 8.59 kcal/FFM, *p* < 0.0001). Nutrient intake was also significantly different between the two groups. Conclusions: Our results suggest that under-reporting of energy intake by collegiate team sport athletes may occur frequently and needs to be taken into consideration in the interpretation of nutrient intake.

## 1. Introduction 

Adequate dietary intake and the correct balance of macronutrients and micronutrients, with appropriate timing to enhance performance and recovery, will enable athletes to train and perform optimally [1]. Team sports share the common feature of intermittent high-intensity activity patterns. However, there is a high variability of game characteristics between sports, between positions and playing styles within the same sport, and from one match to the next. An earlier review by Holway and Spriet [2] found that athletes competing in team sports commonly do not meet recommended dietary intake needs [2,3]. The International Olympic Committee released a statement on sports nutrition in 2010 [4] and nutritional intake guidelines for athletes in 2016 [5] in order to satisfy the diversity of physiological challenges and nutritional needs for team sport athletes [2,6]. 

While there is a unanimous agreement on the need to design and implement nutrition-specific intervention programs for team sport players, there is scarce information on the eating habits and correlations of the nutritional behavior of these players. Understanding the influence of these factors is essential for the design and implementation of effective dietary and nutrition education programs, in order to optimize performance and health through nutrient intake. Several authors tried to assess the team’s food intake by using different dietary assessment methods [7,8,9]. These studies used dietary records, Food Frequency Questionnaire (FFQ), 24h Recall, and qualitative interviews; the studies showed inadequate energy intake that required nutrition education intervention. However, the occurrence of misreporting was not considered.

All methods measuring dietary intake are hampered by errors in precision (repeatability, reliability, reproducibility) and validity (accuracy) [10]. Energy intake (EI) misreporting is characterized by reports of EI habits that are implausibly low or high, that is, under-reporting (UR) or over-reporting (OV), respectively, when compared to the energy requirements estimated using objective methods of energy expenditure, such as the doubly labeled water (DLW) [11]. Both underestimation and overestimation may occur, however, depending on the methodological approach used and the characteristics of the subjects. In general, reporting energy intake may be influenced by factors including age, sex, body fat, body mass index (BMI), education level, social desirability and income level [12,13,14,15]; the bias usually tends to be towards underestimation of habitual energy consumption [16]. 

Under-reporting of dietary intake can be explained by under-recording (intentional or unintentional omission of reporting of some of the food consumed) and/or under-eating (intentional or unintentional reduction of food intake during the study period). 

A review of the published studies using DLW to validate self-reported energy intake in athletes reveals that under-reporting accounts for approximately 10–50% of total energy expenditure, and is mainly due to under-recording rather than under-eating [17]. However, an increased subject burden and/or low self-awareness with regards to food intake may result in under-eating [18]. 

The presence of systematic bias and various types of misreporting in dietary intake assessments may have a big influence on the findings of the associations under study [19]. This problem may also lead to an inadequate estimation of the prevalence of nutrient deficiencies. For that reason, intentional dietary misreporting represents a major concern in studies that monitor dietary intake at the population level and/or evaluate diet–health associations [20,21].

Identification of misreporting in any of its forms and its characteristics is thus crucial to the appropriate interpretation of nutritional data and can be evaluated using criteria developed by Goldberg and Black [22]. The Goldberg method [22] uses predicted basal metabolic rates (BMR) and the ratio of reported energy intake to BMR to estimate the amount of energy available for the activity. 

No studies have been conducted on the dietary habits of Italian collegiate team sport athletes yet. The aim of this study was to assess the dietary intake of a sample of collegiate team sport athletes focusing on the detection of misreporting. Moreover, the characteristics of under-reporters compared to adequate energy reporters were also compared.

## 2. Materials and Methods 

### 2.1. Participants

A total of 50 athletes participated in the study. We involved two teams of volleyball players (13 females and 10 males), two teams of soccer players (19 males) and one team of synchronized swimming players (eight females). All athletes were actively training and competing at the time of the study. The athletes who were managing weight were excluded from the study.

### 2.2. Ethical Approval 

The experimental procedures were approved by the Institutional Ethics Committee of the University of Pavia (the code is 5/2013). Everyone provided written consent to participate in the present study and coach consent was also obtained. The study was carried out according to the Declaration of Helsinki.

### 2.3. Anthropometric and Body Composition Assessments

Anthropometric and body composition measurements included weight, height, body mass index (BMI), skinfold thickness measurement, waist and hips circumference, fat mass and fat free mass. Body weight was measured to the nearest 0.1 kg with a standard balance beam scale (Wunder), whereas body height was measured to the nearest 0.5 cm (Wunder). BMI was calculated by dividing the body weight in kilograms by height in square meters (kg/m^2^) and was additionally categorized according to the WHO classification [23]. The measurement of skinfold thickness was performed three times at the triceps, biceps, subscapular, and supra-iliac sites on the non-dominant side of the body [24] using a Holtain caliper (Holtain Ltd., Crymych, UK). Waist circumference was measured to the nearest 0.1 cm with a measuring tape placed at the midpoint between the lower border of the ribs and the upper border of the pelvis. Bioelectrical impedance measurements were performed using a tetrapolar, multi-frequency bioelectrical impedance analysis (BIA) system (Human-IM DIP, DS-Medigroup, Milan, Italy) at 50 kHz. Subjects laid down with upper and lower arms were slightly abducted to position the electrodes. The Deurenberg’s equation [25] was used to derive body fat, while the fat free mass (FFM) was obtained by the difference in body weight.

### 2.4. Dietary Intake Assessment

Each athlete filled in food records for seven days (three days of training, three days of rest and one match day). A food diary is a detailed daily record of the food and drink that one consumes over a certain period of time, typically used to track calorie consumption or to identify habitual eating patterns. Instructions were provided to record the type and amount of food and drink consumed and to estimate portion sizes. Information on quantities was also collected using household measurements and a photographic atlas of food portions [26]. Additionally, participants were asked to record all supplements taken. Each food record was reviewed for completeness of the information by the first author. Any questions, ambiguities, or omissions regarding the type and amount of food and beverages consumed were clarified with individual athletes via direct interviews. The average kilocalorie, macro and micronutrients content for each athlete’s diet was calculated using the Dieta Ragionata software (version 7.0). Since the main goal of this study was to evaluate the adequacy of the total energy intake, nutrient intake data were analyzed without considering the possible supplements consumed by athletes. 

Recommended intakes of energy and nutrients were based on guidelines outlined in the American College of Sports Medicine, Academy of Nutrition and Dietetics, and Dietitians of Canada Joint Position Statement on Nutrition and Athletic Performance [5]. Energy availability (EA), defined as the dietary intake minus exercise energy expenditure normalized to fat free mass (FFM), is the amount of energy available for the body to perform all other functions after the cost of exercise is subtracted. The EA of 45 kcal/kg/d of FFM was found to be associated with energy balance and optimal health. Athletes who engage in moderate-to-high intensity exercise are advised to consume between 6–10 g/kg of carbohydrates per day. The amount of dietary protein believed to be necessary to support metabolic adaptations, repair, remodeling, and protein turnover is between 1.2–2.0 g/kg/day, spread throughout the day. Fat intakes associated with eating styles that meet dietary goals typically range from 20–35% of the total energy intake. 

As for micronutrients recommendations, the American College of Sports Medicine, Academy of Nutrition and Dietetics, and Dietitians of Canada Joint Position Statement on Nutrition and Athletic Performance [5] suggested to refer to public health guidelines. Therefore, we compared the micronutrients intakes of our study population with the reference intake of nutrients and energy for the Italian population [27].

### 2.5. Relative Energy and Nutrient Intake Assessment

**Figure 1:**
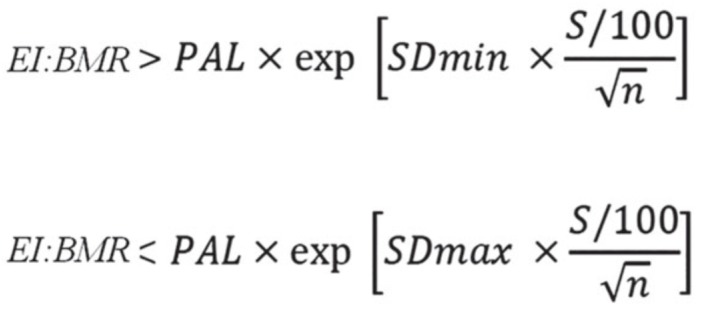
Goldberg/Black equation to derive cut-off values for evaluation of misreporting of energy intake [22]. Legend: EI: Energy intake; BMR: Basal metabolic rate; PAL: Physical activity level; S: Coefficient that considers the variation in EI, BMR and PAL; SDmin: −2 for the 95% lower confidence limit; SDmax: +2 for the 95% upper confidence limit; n: the number of subjects evaluated.

The reported energy intake (EI) was considered in relation to the predicted basal metabolic rate (BMR) and expressed as the ratio of EI to BMR. BMR was estimated (BMRestd) for individual subjects using the Schofield Equation [28] based on gender, age, and weight. Total energy requirements can be estimated by multiplying BMR with physical activity level (PAL). In stable weight conditions, the ratio of EI/BMRestd is equal to the ratio of the total energy expenditure (TEE) to BMRestd. Therefore, in the present analysis, the ratio of EI/BMRestd was used to evaluate the reported energy intake following the procedures outlined by Goldberg et al. [22]. Under-reporting of the energy intake was determined using cut-off values for EI/BMRestd based on the sample size and number of days of the recorded intake. To derive cut-off values for EI misreporting, according to Goldberg et al. [22] we applied the equations reported in Figure 1 at an individual level to determine the rate of under-reporters, plausible reporters and over-reporters [29].

**Figure 2:**
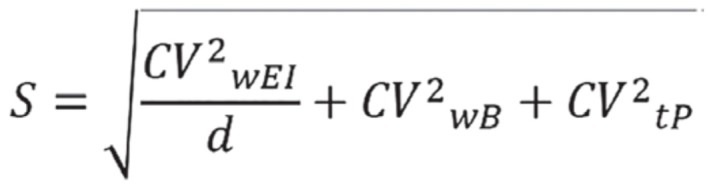
Equation used to calculate the coefficient (S) considering the variation in energy intake, BMR and PAL in the Goldberg/Black equation [22]. Legend: CVwEI: Within-subject variation in energy intake; d: Number of days of diet assessment; CVwB: Within-subject variation in repeated BMR measurements or precision of estimated BMR estimated compared with measured BMR; CVtP: Within-subject variation in PAL.

In the equations, SDmin was −2 for the 95% lower confidence limit; SDmax was +2 for the 95% upper confidence limit and *n* = 1 because we evaluated misreporting at the individual level [30]. According to the FAO/WHO/UNU [31] and SACN [32], the entire sample was classified as “moderately active”, leading to a PAL value of 1.8. In the equations, S was the factor that considered the variation in EI, BMR and PAL [29] and was reported in Figure 2. To calculate S for seven days of food consumption (*d* = 7, by food diary) we used revised factors of Black, considering 23% of within-subject variation for EI (CVwEI), 15% of between-subject variation for PAL (CVtP) and 8.5% of within-subject variation for estimated BMR (CVwB) [29,33]. 

Therefore, subjects with calculated values of the ratio EI: BMRestd in the interval 1.23–2.65 were classified as “plausible or adequate energy reporters”. Subjects with individual EI: BMRestd <1.23 were categorized as “under-reporters or low energy reporters”, subjects with individual EI: BMRestd >2.65 were categorized as over-reporters.

### 2.6. Statistical Analysis

Means and standard deviations were used to describe the characteristics of the sample population. The participants’ carbohydrate and protein intakes were compared with the current minimum sports nutrition recommendations using the one-sample *t*-test (test value of 5 g/kg for carbohydrates and 1.2 g/kg for protein) [5,30,34,35]. The paired-sample *t*-test was used to compare the participants’ reported energy intake with their BMRestd. The proportion of participants meeting EI/BMRestd >1.23 was calculated. The independent *t*-test was used to test differences between group means. Bivariate Pearson correlation coefficients were calculated between anthropometric characteristics, average energy intake, and EI/BMRestd. The SPSS 15.0 was used for statistical assessment.

## 3. Results

Table 1 shows the demographic and anthropometric characteristics of the participants of the study. The mean age was 21.32 ± 4.16 years and the majority of these were male (*n* = 29). The mean BMI was 22.40 ± 2.99 kg/m^2^ and the body fat percentage was 23.53 ± 5.85%, and the fat free mass was 52.76 ± 12.41 kg. Mean Body Mass Index (BMI) in male athletes was 23.40 ± 3.20 kg/m^2^ and the fat mass (FM) was 20.80 ± 5.98%; in particular, in the soccer team the mean BMI was 23.26 ± 1.92 kg/m^2^ and the fat mass in percentage was 19.75 ± 3.74%, while in volleyball players the mean BMI was 23.67 ± 4.92 kg/m^2^ and the FM was 22.80 ± 8.74%. In female athletes the mean BMI was 21.00 ± 2.03 kg/m^2^ and the fat mass was 27.30 ± 2.85, in particular, volleyball team athletes had 20.91 ± 2.14 kg/m^2^ of BMI and the FM percentage was 26.95 ± 3.31%; while in the synchronized swimming team the mean BMI was 21.14 ± 1.95 kg/m^2^ and the FM was 27.89 ± 1.95%. Considering the whole sample, energy intake/kg FFM was 40.01 ± 10.64 kcal/kg body weight, protein intake/kg body weight was 1.16 ± 0.31 g/kg body weight, carbohydrate intake/kg body weight was 3.91 ± 1.14 g/kg body weight and fat intake was 35.04 ± 8.19% energy intake.

According to cut-off values for under-reporting 28 out of 50 athletes, (56%) were classified as low energy reporters. Nobody was classified as over-reporting. The characteristics of low and adequate energy reporters are compared in Table 2. 

The low energy reporters (16 M/12 F) had significantly higher BMI (23.17 ± 3.46 kg/m^2^ compared to 21.41 ± 1.91 kg/m^2^, *p* = 0.038) than the adequate energy reporters (Table 2). Total energy intake (1703.65 ± 382.17 kcal compared to 2444.45 ± 347.55 kcal, *p* < 0.0001), energy intake/kg body weight (BW) (24.73 ± 4.83 kcal/kg BW compared to 37.77 ± 5.79 kcal/kg BW, *p* < 0.0001) and energy intake/kg fat free mass (FFM) (33.34 ± 6.56 kcal/FFM compared to 48.51 ± 8.59 kcal/FFM, *p* < 0.0001) were significantly lower in low energy reporters than the adequate energy reporters. 

Nutrient intake was also significantly different between the two groups (Table 3). Protein intake/kg body weight (0.98 ± 0.21 g/kg BW compared to 1.39 ± 0.27 g/kg BW, *p* < 0.0001), carbohydrate intake/kg body weight (3.29 ± 0.91 g/kg BW compared to 4.71 ± 0.89 g/kg BW, *p* < 0.0001) and fat intake (33.02 ± 8.65% compared to 37.60 ± 6.91%, *p* = 0.048) were significantly lower in low energy reporters than the adequate energy reporters (13 M/9 F). The fiber intake was not different between the two groups. As for micronutrients intake, calcium (*p* = 0.001), phosphorus (*p* = 0.001), zinc (*p* = 0.025), thiamine (*p* = 0.037) and riboflavin (*p* = 0.021) were significantly lower in low energy reporters than in the adequate energy reporters.

There wasn’t any correlation between anthropometric characteristics, average energy intake, and EI/BMRestd.

## 4. Discussion

Our results confirm a high percentage of under-reporting among team sport collegiate athletes even if this percentage is lower than what was reported in previous studies conducted in team sport athletes such as soccer, rugby, and artistic gymnastics [36,37,38]. These percentages of under-reporting in our study and in previous ones on team sport athletes are higher than the under-reporting frequency in the general population [39,40]. Kobe et al. [39], examining under–reporting of energy intake among Slovenian adolescents entering high school, described UR proportions of 34% for boys and 27% for girls, respectively. A similar percentage (27%) was reported by Ouelette et al. [40] in sixty subjects (40 women and 20 men) recruited from the University of Connecticut. The phenomenon must be taken into consideration when interpreting the results of dietary surveys. The omission would lead to erroneous interpretation of results. Despite the individual variability for energy, macro, and micronutrients requirements, in general, the athletes reported an insufficient mean intake of energy, protein, carbohydrate and fiber, and an adequate mean fat intake consumption compared to recommendations [5]. Excluding under-reporters from the analysis, the mean nutrient intake is adequate for proteins (1.39 ± 0.27 g/kg BW), insufficient for carbohydrates and fiber, and excessive for fat compared to recommendations [5]. As for mean micronutrient intakes, Calcium, Potassium, Iron and Zinc are inadequate compared to recommendations [27].

Deficiencies in energy and nutrients can have implications for an athlete’s performance including a loss of fat free mass, disturbances to immune function, decreased bone mineral density, increased susceptibility to injury and increased prevalence of symptoms of overtraining [1]. Unbalanced intakes are described in other studies conducted in team sport athletes [8,38,41] and suggest the need of nutrition education intervention. These interventions should consider that - due to the sport-specific factors, physique and position differences - dietary advice for team sport athletes should be individualized.

Under-reporters were characterized by a higher BMI than adequate reporters. Previous research has indicated that the degree of misreporting increases with increasing body mass, specifically adiposity [42,43] and BMI [44]. 

The possible causes of under-reporting can be: alteration of the usual intake during the period of monitoring; inaccurate reporting of food intake, to improve the perception of what the athlete is eating (i.e., omission or underestimation of food or meals considered undesirable, or false reporting of foods considered desirable); and erroneous quantification or description while reporting food intake [45]. 

For example, under-reporting can be due to the unconscious omission of eating occasions, recording fatigue or conscious misreporting (e.g., denial of consumption) [46], further suggesting that especially missing eating episodes contribute to under-reporting [47]. An alteration of the usual intake during the monitoring period might have caused a decrease in consumption or even an exclusion of certain types of food, to show a faultless diary [11]. Poppitt et al. [48] found that although the main meals were well reported, between-meal snacks were omitted from the participants’ 24-h report, with more than one third of snack consumption being absent. Similarly, Johansson et al. [44] found that under-reporters (relative to their food intake level) seem to selectively under-report unhealthy snacks (while that’s less likely with healthy foods). Certain types of foods or eating occasions are more likely to be misreported than others, either due to the inconvenience of reporting (e.g., snacks), failure to recognize that they represent intake (e.g., foods and drinks consumed during exercise) or the desire to appear to eat better than in reality (e.g., reduction in high fat and sugary foods, increase in fruits and vegetables) [45]. In addition, factors such as high energy requirements, eating away from the home environment, applicability of ‘standard’ portion sizes, and the wide use of commercial sports foods, drinks and supplements can make it more difficult to quantify food intake [18,49]. The portion sizes that were described might have been inaccurate, especially by those who did not weigh food and/or did not know common household measures, which might have led to an underestimation of the true intakes [50,51]. 

When trying to explain some of the under-reporting in sport college students, it is necessary to consider the fact that most of them are often exposed to extra pressures (i.e., exams, lessons and training), that can negatively influence their dietary habits and the available time to accurately compile the diary. On this point, Caccialanza et al. describing under-reporting in 43 young high level soccer players, took into account the fact that during the school term, training begins straight after school, which would affect meal time patterns [37]. Moreover, psychosocial factors, such as increased emphasis on leanness or the desired weight loss, can influence under-reporting. Jonnalagadda et al. [36] described under-reporting in 61% of elite female gymnasts, underlining that individual psychological profile, dieting behavior and perceived quality of foods may be important determinants of the degree of under-reporting.

Well-designed studies on dietary assessment in athletes often provide inconsistent findings. Over the years, the discussion has evolved on whether this could also, at least partially, reflect limitations of the dietary assessment methods that generate measurement errors of different magnitude. Kipnis et al. [52] described two potential components of the dietary measurement errors. The first one reflects the correlation between the error and true intake (“intake-related” bias), while the second is independent of the true intake and represents errors related to the participant’s personal characteristics (“person-specific” bias). Measurement errors can be systematic or random. Systematic errors reflect methodological weaknesses, appear at the group level, and generate various misclassification. Random errors occur at the individual level, generate non-differential misclassification, and generally influence relative risk estimates and reduce statistical power to detect them [53]. Random errors during measurements on any given day can be prevented or minimized by incorporating standardized quality control procedures. 

Another important aspect to consider is the duration of the food diary: an increase in the number of days of recording increases the likelihood that it will represent the usual intake, but reduces the compliance of the subject in keeping an accurate record. Few studies have examined the optimal recording period for athletes by looking systematically at issues of recording compliance or day to day variability in food/nutrient intake. Professional experience suggests that some athletes are diligent, or even too meticulous, at record keeping; they are accustomed to measuring aspects of their life in accurate detail and motivated by the idea that the activity could lead to better performance outcomes. Such individuals may be able to record a seven-day food diary with little burden and careful precision. In contrast, other athletes are poor candidates for food diaries as an overcommitted lifestyle can leave little time or enthusiasm for real-time recording of food intake, while the imposition by a coach of an effort-requiring activity when the athlete is disinterested in nutrition is unlikely to achieve a useful outcome [45]. We have used a seven-day food diary that seems to be an adequate analysis period, a perspective method of recording dietary intakes, it provides confidence in the accuracy of quantified information and it can assess how well patterns of food/fluid intake track with the changing needs of workouts and competitions [45]. Moreover, this dietary assessment may also be considered as an instrument to make students aware of their choices and help them improve their habits.

A recent review [54] suggests that a combined dietary data collection method (i.e., Food Record and 24-h Dietary Recalls) may prove to be effective in quantifying energy intake in athletes.

Finally, emerging image-assisted technical innovations such as wearable cameras, handheld devices, and mobile telephone technology have been shown to improve participant compliance by reducing the burden of recording, as well as enhancing the accuracy of the data recorded [55,56,57,58].

In our study we adopted the Goldberg cut-off [22], which looks at the reported energy intake relative to the measured or predicted basal metabolic rate to identify implausible habitual eating patterns and thus significant misreporting in a food record [59]. The Goldberg cut-off can be used to evaluate the mean population bias in the reported energy intake, but information on the activity or lifestyle of the population is needed to choose a suitable PAL energy requirement for comparison. Sensitivity for identifying under-reporters at the individual level is limited. In small studies such as ours, it is desirable to measure energy expenditure with indirect calorimetry, or to calculate individual energy requirements, and to compare energy intake directly with energy expenditure.

A limitation of the present study is the use of prediction equations for estimating BMR and energy needs. In these data, athletes with actual BMRs that are lower than the predicted values might be miscategorized as under-reporters, since they would actually be consuming a level of energy that meets their current requirements. However, since a lower BMR than the prediction may result from under-consumption of energy, using the predicted value may be useful in identifying under-reporters and under-consumers. There is limited information on the validity and applicability of existing formulas for determining energy requirements of various subgroups of the population, such as team sport athletes and individuals with high levels of physical activity.

## 5. Conclusions

Our study is the first to examine under-reporting of energy intake in Italian collegiate team sport athletes and the differences in body composition, in macro and micronutrient intakes of under-reporters compared to adequate energy reporters.

Our results suggest that under-reporting of energy intake by the collegiate team sport athletes may occur frequently. This can have an impact on their reported macro and micro nutrient intake and cause misleading interpretation of nutritional risk by researchers and practitioners. Future research needs to better understand the causes of misreporting in dietary intake studies and develop specific assessment methods to prevent or correct the phenomenon.

## Figures and Tables

**Table 1:** Anthropometric, body composition characteristics and mean daily nutrient intake of the sample population (Mean ± DS).

	Total (*n* = 50)	Male (*n* = 29)	Female (*n* = 21)
Age (years)	21.32 ± 4.16	22.86 ± 3.59	19.19 ± 4.02
Weight (kg)	68.06 ± 13.91	75.91 ± 12.71	57.21 ± 5.88
BMI (kg/m^2^)	22.40 ± 2.99	23.40 ± 3.19	21.00 ± 2.03
BMRestd (kcal/day)	1662.74 ± 262.12	1834.70 ± 194.23	1425.26 ± 115.84
EI/BMRestd	1.22 ± 0.28	1.22 ± 0.23	1.21 ± 0.29
FM BIA (%)	23.53 ± 5.85	20.80 ± 5.98	27.31 ± 2.85
FFM (kg)	52.76 ± 12.41	61.77 ± 7.91	40.33 ± 3.15
Energy intake (kcal/day)	2029.60 ± 519.83	2233.74 ± 482.03	1747.70 ± 439.19
Energy intake (kcal/kg/day)	30.47 ± 8.36	29.93 ± 7.34	31.21 ± 9.74
Energy intake (kcal/FFM/day)	40.01 ± 10.64	37.24 ± 8.20	43.84 ± 12.52
Carbohydrate (g/kg/day)	3.91 ± 1.14	3.91 ± 1.20	3.91 ± 1.09
Protein (g/kg/day)	1.16 ± 0.31	1.17 ± 0.32	1.14 ± 0.31
Fat (%/day)	35.04 ± 8.19	34.84 ± 8.67	35.30 ± 7.67
Fiber (g/day)	11.96 ± 5.29	14.18 ± 5.32	8.90 ± 3.46

Note. EI/BMRestd = ratio of energy intake to BMR estimated using the Schofield equation. Abbreviations: BMI: Body mass index; BMRestd: Basal metabolic rate estimated; EI: Energy intake; FM: Fat mass; BIA: bioelectrical impedance analysis; FFM: Fat free mass.

**Table 2:** Anthropometric and body composition characteristics of athletes classified as low and adequate energy reporters based on the ratio of energy intake to the estimated basal metabolic rate (Mean ± DS).

	Low Energy Reporters (EI/BMRestd < 1.23) (*n* = 28)	Adequate Energy Reporters (EI/BMRestd > 1.23) (*n* = 22)
Sex (M/F)	16/12	13/9
Age (years)	22.11 ± 4.48	20.32 ± 3.56
Weight (kg)	69.93 ± 15.96	65.67 ± 10.64
BMI (kg/m^2^)	23.17 ± 3.46	21.41 ± 1.91 *
BMRestd (kcal)	1672.68 ± 302.34	1650.08 ± 206.13
EI/BMRestd	1.02 ± 0.15	1.48 ± 0.16 *
FM BIA (%)	24.66 ± 6.24	22.10 ± 5.09
FFM (kg)	52.99 ± 13.07	52.47 ± 11.82

* Significant differences between the two groups, *p*-value < 0.05. Abbreviations: BMI: Body mass index; BMRestd: Basal metabolic rate estimated; EI: Energy intake; FM: Fat mass; BIA: Bioelectrical impedance analysis; FFM: Fat free mass.

**Table 3:** Mean daily nutrient intake of athletes classified as low and adequate energy reporters based on the ratio of energy intake to the estimated basal metabolic rate (Mean ± DS).

	Low Energy Reporters (EI/BMRestd < 1.23) (*n* = 28)	Adequate Energy Reporters (EI/BMRestd > 1.23) (*n* = 22)
Energy intake (kcal/day)	1703.65 ± 382.17	2444.45 ± 347.55 *
Energy intake (kcal/kg/day)	24.73 ± 4.83	37.77 ± 5.79 *
Energy intake (kcal/FFM/day)	33.34 ± 6.56	48.51 ± 8.59 *
Carbohydrate (g/kg/day)	3.29 ± 0.91	4.71 ± 0.89 *
Protein (g/kg/day)	0.98 ± 0.21	1.39 ± 0.27 *
Fat (%/day)	33.02 ± 8.65	37.60 ± 6.91 *
Fiber (g/day)	10.82 ± 5.20	13.42 ± 5.15
Calcium (mg/day)	448.68 ± 225.75	767.81 ± 284.75 *
Phosphorus (mg/day)	903.87 ± 242.39	1256.98 ± 334.08 *
Potassium (mg/day)	2185.40 ± 799.23	2379.63 ± 907.26
Sodium (mg/day)	1572.97 ± 671.87	2049.23 ± 988.67
Iron (mg/day)	9.89 ± 3.75	10.69 ± 3.88
Zinc (mg/day)	5.86 ± 1.74	7.61 ± 2.62 *
Thiamine (mg/day)	0.97 ± 0.46	1.38 ± 0.65 *
Riboflavin (mg/day)	1.05 ± 0.46	1.48 ± 0.59 *
Niacin (mg/day)	15.25 ± 6.07	19.41 ± 10.01
Vitamin A (µg RE/day)	740.87 ± 965.65	713.76 ± 474.57
Vitamin C (mg/day)	128.56 ± 98.52	109.07 ± 68.39

* Significant differences between the two groups, *p*-value < 0.05. Abbreviations: BMRested: Basal metabolic rate estimated; EI: Energy intake; FFM: Fat free mass; RE: Retinol equivalent.
